# Perceived Stress and Smartphone Application-Based Addiction Among Chinese Young Adults: The Mediating Role of Psychological Distress and the Moderating Role of Self-Control

**DOI:** 10.11621/pir.2025.0407

**Published:** 2025-12-01

**Authors:** Jia Guo, Ying Xie, Fangfang Zheng, Feifei Wang

**Affiliations:** a Chongqing Business Vocational College, Chongqing, China; b Army Medical University, Chongqing, China

**Keywords:** perceived stress, Smartphone application-based addiction, self-control, psychological distress, young adults

## Abstract

**Background.:**

Previous research has established the significant role of perceived stress in contributing to Smartphone addiction, but the specific effects and mechanisms through which perceived stress influences smartphone application-based addiction (SABA) among young adults remain insufficiently understood.

**Objective.:**

This study investigates the relationship between perceived stress and the smartphone application-based addiction (SABA) in emerging adulthood, focusing on the mediating role of psychological distress and the moderating role of self-control.

**Design.:**

We conducted a cross-sectional survey of 1.911 young adults in Southwest China, utilizing the Perceived Stress Scale (PSS-4), the Smartphone Application-Based Addiction Scale (SABA), the Patient Health Questionnaire-4 (PHQ-4), and the Brief Self-Control Scale (BSCS).

**Results.:**

Perceived stress positively predicted psychological distress (β = .47, *t* = 24.38, *P* < .001), psychological distress positively predicted Smartphone application-based addiction (β = .42, *t* = 7.04, *P* < .001), and perceived stress positively predicted smartphone application-based addiction (β = .65, *t* = 11.46, *P* < .001). Psychological distress was a mediating variable between the relationship between perceived stress and smartphone application-based addiction, with a mediating effect size of .19 (95% CI = [.13, .26]), accounting for 22.62% of the total effect. Self-control moderated the relationship between perceived stress and psychological distress in young adults (β = -.03, *t* = -7.09, *P* < .001).

**Conclusion.:**

Our findings reveal a positive correlation between perceived stress and SABA, with psychological distress serving as a mediator. The impact of perceived stress on SABA is more signiicant among individuals with lower self-control, indicating that targeted interventions for these groups may be particularly beneicial.

## Introduction

In recent years, the widespread use of smartphones has profoundly affected people’s daily lives. Among young adults, smartphones are not only the main communication tool, but also a source of entertainment, education and socialization (Irimiás et al., 2021). The numerous advantages offered by smartphones are indisputable; nevertheless, concerns regarding the potential risks of addiction arising from overuse have emerged (Chen et al., 2016), particularly among young adults and college students in China. Recent studies indicate smartphone addiction is on the rise, with China among the countries showing the highest levels (Olson et al., 2022). This phenomenon is amplified by China’s unique digital ecosystem, which is characterized by a “mobile-first” trajectory that leapfrogged the personal computer era, and the dominance of “super-apps” like WeChat and Alipay. Unlike the fragmented application landscape in many Western countries, these platforms integrate social networking, e-commerce, inancial services, and entertainment into a single, highly cohesive environment, fostering unprecedented levels of user engagement and dependency. The intense market competition also drives developers to employ aggressive user retention strategies, such as gamification and persistent notifications, which may exacerbate addictive tendencies. A growing body of research has demonstrated a correlation between increased use of smartphones and social media, and an increase in psychological distress, including symptoms of depression and anxiety, as well as sleep disorders (Geng et al., 2021; Li et al., 2020; Sohn et al., 2019).

We operationalize Smartphone Application-Based Addiction (SABA) as a content-centric phenomenon characterized by compulsive engagement with specific applications (e.g., social media, gaming, short-video platforms), distinct from general smartphone addiction’s device-centric focus on overall usage metrics. Th is distinction aligns with Song et al.’s framework where SABA manifests through application-specific behavioral patterns rather than mere screen time duration (Song & Zhao, 2025). This aligns with the components of addiction measured by the SABAS scale, such as salience, mood modiication, and tolerance, which are driven by the functions and feedback loops within applications themselves (Csibi et al., 2018). Therefore, studying SABA allows for a more nuanced understanding of the psychological mechanisms, as the addictive behavior is tied to specific gratifying activities (e.g., social validation, escapism) rather than to the phone in general.

Emerging adulthood is a period of heightened life transitions and stress, making individuals vulnerable to maladaptive coping strategies like smartphone overuse (Lane et al., 2017). Perceived stress has been identified as a significant risk factor for smartphone addiction (Vujic & Szabo, 2022). Th eoretical frameworks such as the stress-coping models suggest that individuals may turn to smartphones as a coping mechanism for stress and negative emotions, inadvertently fostering addictive behaviors (Kardefelt-Winther, 2014). Recent research has also highlighted the novelty of examining smartphone application-based addiction, distinguishing it from general smartphone use by focusing on speciic app-driven compulsive behaviors, which offers a fresh perspective in the field (Zhang et al., 2025). Moreover, cross-cultural analyses reveal unique patterns in Chinese populations, where the direct efect of mobile phone addiction on perceived stress is more pronounced compared to other groups, potentially due to varying degrees of addiction severity and cultural expectations (Liu et al., 2022). This underscores the need for context-specific research, particularly in China, where academic stress and societal pressures may amplify vulnerability to addiction (Shen et al., 2021).

### Perceived Stress and Smartphone Addiction

Perceived stress refers to the degree to which individuals perceive their lives as unpredictable, uncontrollable, and overloaded (Cohen et al., 1983), which can have a range of amplitude effects on psychological and behavioral health, such as insomnia (Veeramachaneni et al., 2019), depressive symptoms (Elayoubi et al., 2023), suicidal behaviors (Chen & Kuo, 2020), and so on. Simultaneously, emerging adults may experience elevated levels of stress and be more susceptible to stress during periods of transition (Varma et al., 2021). With the development of society and the popularization of smartphones, constant stress for young adults has become the new normal.

Research on stress-coping models and smartphone addiction reveals a complex relationship mediated by various psychological factors. Perceived stress is positively correlated with smartphone addiction (AlSaif et al., 2023; Hong et al., 2021). Problem-focused coping and self-efficacy are negatively associated with smartphone addiction, while emotion-focused and avoidance coping are positively related (Flynn et al., 2020).While smartphones can be used as a coping mechanism, excessive use may lead to dependency and exacerbate stress. Th erefore, the relationship between perceived stress and the smartphone application-based addiction needs to be further clariied. We proposed the following hypothesis:

*HI: Perceived stress has a positive relationship with SABA among young adults*.

### Psychological Distress as a Mediator

Psychological distress is deined as an emotionally distressing state experienced by an individual in response to a challenging or traumatic life event (Ridner, 2004). Research indicates that psychological distress, particularly depression and anxiety, is widespread among young adults (Baxter et al., 2016; Glowacz & Schmits, 2020; Whiteford et al., 2013). Furthermore, psychological distress serves as a mediator between adverse experiences and negative health outcomes. Psychological distress has been found to predict smartphone addiction (Kayî§, 2022; Law & Yap, 2022), while smartphone addiction also predicts psychological distress (Lian et al., 2021), suggesting a bidirectional relationship. Higher levels of perceived stress correspond to intensiied psychological distress, which encompasses ailments like depression, anxiety, and lower self-affirmation (Shruthi et al., 2023; Wang & Wang, 2019). Different aspects of smartphone use, such as problematic use, phone checking, and screen time, have distinct relationships with psychological distress (Tng & Yang, 2024). Research suggests that psychological distress plays a complex role in addictive behaviors, acting as both an antecedent and consequence. While a positive correlation between perceived stress and SABA is established, few studies examine how psychological distress channels this relationship.

Perceived stress and psychological distress are closely related yet distinct constructs. Th eoretically, perceived stress refers to the subjective cognitive appraisal of life events as unpredictable, uncontrollable, or overwhelming, often measured as a global perception without specifying emotional outcomes (Cohen et al., 1983). In contrast, psychological distress encompasses the emotional and somatic responses to stressors, including symptoms of anxiety, depression, and overall emotional suffering, reflecting a more affective and symptomatic state (Ridner, 2004). Methodologically, this study employs the PSS-4 to capture perceived stress as a broad evaluative process, while the PHQ-4 assesses psychological distress through specific symptom-based items on anxiety and depression over the prior two weeks. Despite their correlation, the distinction is evident in their roles: perceived stress initiates a cognitive pathway that can lead to distress, which then mediates maladaptive behaviors like SABA. This mediation is grounded in Stress and Coping Theory, where unaddressed stress appraisals escalate into distress, prompting coping via smartphone apps (Lazarus & Folkman, 1984). Therefore, we proposed the following hypothesis:

*H2: Psychological distress has a mediating effect on the relationship between perceived stress and SABA*.

### Self-Control as a Moderator

Self-control is the ability to overcome impulsive, habitual, or automated responses and consciously take charge of the direction of one’s behavior (Baumeister et al., 2007; Yu et al., 2013), which plays an important role in controlling stress, psychological distress, and smartphone addiction. According to the self-control power model, executing self-control requires a limited supply of cognitive resources (Baumeister et al., 2007). Insufficient self-control can lead to behavioral and impulse control problems. Furthermore, self-control directly impacts smartphone dependency and also moderates the inluence of additional risk factors (for example, psychological distress). For example, studies have found that self-control can moderate the relationship between psychological distress and food addiction in college students (Luo et al., 2022). Self-control has a small to moderate positive efect on behaviors in many areas (De Ridder et al., 2012), showing that there are both mediation and moderation efects in many health-related actions. Moreover, the speciic stage at which self-control intervenes — whether in mitigating the emotional impact of stress or directly influencing addictive behaviors — remains unclear.

To address this, we position self-control as a moderator between perceived stress and psychological distress, grounded in Self-Regulation Theory, which suggests that self-control is a critical resource for managing emotional responses to stress before they escalate into maladaptive behaviors. We argue that lower self-control amplifies the transformation of stress into distress. This focus is crucial as it highlights an early intervention window where enhancing self-control could prevent stress from manifesting as distress and subsequent addiction, a perspective less explored compared to direct behavioral moderation. Th erefore, we proposed the following hypothesis:

*H3: Self-control is a moderator between perceived stress and psychological distress*.

### The Present Study

In summary, the complex psychological pathways through which stress influences smartphone application-based addiction (SABA), as well as the boundary conditions that modulate this relationship, remain insufficiently explored in existing research. To address these lacunae, this study proposes a novel moderated mediation model to elucidate the relationship between perceived stress and SABA among young adults in China.

Th e significance of this study lies in its integrated theoretical framework. First, we move beyond simple correlation by hypothesizing that psychological distress serves as a pivotal mediator, channeling the effects of perceived stress into addictive behaviors. This extends the Stress and Coping Theory by providing a specific mechanism, which aligns with Self-Regulation Th eory, suggesting that individuals may engage with smartphone applications as a way to self-soothe or escape from negative emotional states brought on by stress. Second, we introduce self-control as a critical moderator to define the boundary conditions of this mediational process. This perspective, grounded in Self-Regulation Theory, posits that individuals with lower self-control are more susceptible to the detrimental efects of stress on their psychological well-being, thus making them more vulnerable to developing SABA. Th is adds significant depth to existing models by identifying a specific at-risk population and highlighting the importance of individual diferences in the development of addictive behaviors.

**Figure 1.**
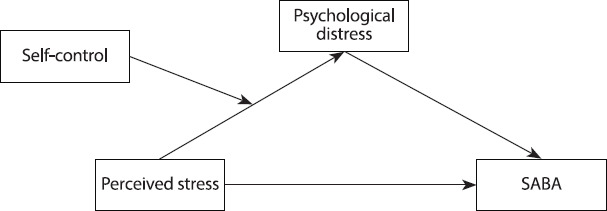
The proposed moderated mediation model

By integrating these components, our model ofers a more comprehensive and nuanced explanation of the psychological mechanisms driving SABA. Th e present study i rst investigated whether psychological distress mediates the relationship between perceived stress and SABA among young adults, and then explored the moderating role of self-control in perceived stress and SABA with psychological distress as a mediator (see *Figure 1*).

## Method

### Participants

This study collected data through an online survey on the website Questionstar (https://www.wjx.cn/). Background information about participants, the Perceived Stress Scale (PSS-4), the Smartphone Application-Based Addiction Scale (SABAS), the Patient Health Questionnaire-4 (PHQ-4), and the Brief Self-Control Scale (BSCS) were included. Th e online survey began with a detailed description of the purpose and requirements of the study, followed by written informed consent. Only after signing an informed consent form could participants take the online survey. Adopting a strategy of convenience sampling, we successfully recruited 2.000 young adults from Southwest China, which resulted in a notable response rate of 95.55%, leading to a concluding group of 1.911 participants aged 18 to 26 years (mean age = 19.89 years; age standard deviation = .78). Th e inquiry involved 1.035 women participants (54.16%) and 876 men participants (45.84%). All participants signed an informed consent form prior to the study’s start and received information about their right to withdraw at any time. The study has been conducted in compliance with the Helsinki Declaration and approved by the Army Military Medical University.

### Measures

#### Perceived Stress Scale (PSS)

The Perceived Stress Scale (PSS) is a widely used instrument designed by Cohen and colleagues (Cohen et al., 1983). The present study used the PSS-4, which is a simplified version of the widely used PSS assessment tool, and the Chinese version has good psychometric properties (Guan et al., 2024). This version measures perceived stress using a 5-point Likert scale (0 (never) to 4 (very oten). Scores range from 0 to 16, with higher scores indicating greater psychological stress. Th e Cronbach’s α coeicient for this scale is .70.

#### Smartphone Application-Based Addiction Scale (SABAS)

The Smartphone Application-Based Addiction Scale is a validated brief instrument developed by Csibi et al. to screen for the risk of Smartphone application-based addiction (Csibi et al., 2018). The six items on the scale are based on the six criteria of the Component Model of Addiction: salience, mood, correction, tolerance, withdrawal conflict, and relapse. The instrument uses a Likert scale from 1 (strongly disagree) to 6 (strongly agree), for a total score of 6 to 36 (Zhang et al., 2022). Higher scores indicate a greater risk of smartphone addiction. The Cronbach’s α coefficient for this scale is .67.

#### Brief Self-Control Scale (BSCS)

The Brief Self-Control Scale (BSCS) is a widely used measure of self-control with seven items comprising two dimensions: self-discipline and impulse control (Morean et al., 2014). This version has been shown to have good psychometric properties in Chinese samples (Tao et al., 2021). The scale is rated on a 5-point Likert scale, with higher scores indicating higher levels of self-control. The Cronbach’s α coefficient for this scale is .86.

#### Patient Health Questionnaire-4 (PHQ-4)

Psychological distress was assessed using the widely used and well-validated Patient Health Questionnaire-4 (PHQ-4), which consists of the i rst two items of the Generalized Anxiety Disorder-7 (GAD-7) and the Patient Health Questionnaire-9 (PHQ-9), which evaluates core criteria for depression and anxiety disorders in participants over the prior two weeks. The PHQ-4 has been applied to Chinese populations, demonstrating good validity and reliability (Xu et al., 2021). The evaluation framework consists of a 4-point scoring range from 0 (not at all) to 3 (almost every day), which yields a cumulative score that can change from 0 to 12, indicating higher levels of distress with elevated scores. Different levels of psychological distress are defined as different scores: normal (0 to 2), mild (3 to 5), moderate (6 to 8), and severe (9 to 12) (Kroenke et al., 2009). The Cronbach’s α coefficient for this scale is .82.

### Data Analysis

This study analyzed common method bias, descriptive statistics, and correlations for all variables using SPSS 24.0. Mediated and moderately mediated models were then analyzed using SPSS’s PROCESS macro (Hayes, 2013). Bias-adjusted 95% conidence intervals (CIs) were derived employing 5.000 bootstrap resampling techniques, wherein conidence intervals that exclude zero signify statistically signiicant efects. Prior to hypothesis testing, multicollinearity among predictor variables was assessed using Variance Inflation Factor (VIF) and Tolerance statistics. VIF values exceeding 10 or Tolerance values below 0.10 were considered indicative of problematic multicollinearity requiring remedial action. First, *Model 4* of the PROCESS macro analysis examined the mediating inluence of psychological distress amid the association of perceived stress with SABA. Subsequently, *Model 7* of the PROCESS macro analysis was used to test the moderating mediating efect, *i.e*., whether self-control moderates the effect of perceived stress on psychological distress (as shown in *Figure 1*).

## Results

### Common Method Variance

In order to avoid common methodological variability, measures such as reverse scoring were used for pre-procedural control of the study design. Harman’s single-factor analysis was employed to detect prevalent methodological biases (Podsakof et al., 2003). The results showed that 4 factors’ eigenvalues were > 1. The first factor loaded 28.77% of all inter-item covariates and accounted for < 40% of all inter-item covariates. This indicates that there is no significant generalized method bias in this study.

### Correlations Among Variables

As shown in *Table 1*, significant correlations were found between perceived stress, psychological distress, SABA, and self-control. Speciically, perceived stress was significantly positively correlated with SABA (*r* = .36, *p* < .001) and psychological distress (*r* = .49, *p* < .001), while it was significantly negatively correlated with self-control (*r* = -.61, *p* < .001). Psychological distress was significantly positively correlated with SABA (*r* = .30, *p* < .001) and significantly negatively correlated with self-control (*r* = -.41, *p* < .001). SABA was signifi cantly negatively correlated with self-control (*r* = -.47, *p* < .001).

**Table 1:** Correlation Between Overall Variables

	M ± SD	1	2	3	4
1. SABA	20.60 ± 5.31	1			
2. Perceived stress	6.52 ± 2.27	.36^a^	1		
3. Psychological distress	2.49 ± 2.17	.30^a^	.49^a^	1	
4. Self-control	22.62 ± 3.42	–.47^a^	–.61^a^	–.41^a^	1

*Note:* ^*a*^ *= p <.001*.

### Multicollinearity Diagnosis

Prior to conducting regression analyses, multicollinearity among predictor variables was assessed using Variance Inflation Factor (VIF) and Tolerance values. Multi-collinearity occurs when predictor variables are highly intercorrelated, potentially leading to unstable regression coefficient estimates and inflated standard errors. The VIF values for all predictor variables were below the commonly accepted threshold of 10, with perceived stress (VIF = 1.767), self-control (VIF = 1.583), and psychological distress (VIF = 1.346) all indicating acceptable levels of multicollinearity. Correspondingly, Tolerance values were above the .10 threshold (perceived stress: .566; self-control: .632; psychological distress: .743), further confirming that multicollinearity did not pose a significant threat to our regression analyses (see *Table 2*).

**Table 2:** Multicollinearity Diagnostics for Predictor Variables

Variable	VIF	Tolerance
1. Perceived stress	1.767	.566
2. Psychological distress	1.346	.743
3. Self-control	1.583	.632

*Notes: VIF = Variance Inflation Factor. VIF values < 10 and Tolerance values > .10 indicate acceptable levels of multicollinearity*.

### A Mediation Model Test of Psychological Distress

This PROCESS macro (*Model 4*) (Hayes, 2013) was used to examine whether psychological distress mediated the relationship between perceived stress and SABA. Ater controlling for gender and age, (see *Table 3*) perceived stress signiicantly and positively predicted SABA (*β* = .84, *p* < .001), thus validating H1.

In addition, perceived stress signiicantly and positively predicted psychological distress (*β* = .47, *p* < .001) and psychological distress significantly and positively predicted SABA (*β* = .65, *p* < .001). Further bias-corrected Bootstrap tests indicated a direct effect of .65 (95% CI = [.54, .76]) and a significant mediating effect of psychological distress with an indirect effect of .19 (95% CI = [.13, .26]). This suggests that perceived stress has a direct efect on SABA, which in turn has an efect on SABA through the partial mediation of psychological distress, with a mediation efect of 22.62% of the total effect (see *Table 4*). This result supports H2.

**Table 3:** A Mediation Model Test of Psychological Distress

Independent variables	Model 1 (SABA)		Model 2 (Psychological distress)		Model 3 (SABA)	
	B	t	β	t	β	t
Gender	.23	1.02	.03	.37	.22	.97
Age	-.29	-1.98^a^	.08	1.43	-.32	-2.23^a^
Perceived stress	.84	16.85^c^	.47	24.38^c^	.65	11.46^c^
Psychological distress					.42	7.04^c^
*R* ^*2*^	.13		.24		.15	
*F*	96.48^c^		199.08^c^		86.61^c^	

*Notes. All variables in the model have been standardized. N = 1911. Gender is a dummy variable, encoding 0 = female and 1 = male. SABA = Smartphone Application-Based Addiction*. ^*a*^ *= p <.05;* ^*b*^ *= p <.01;* ^*c*^ *= p < .001*.

**Table 4:** Analysis of the Mediating Effects of Psychological Distress

	Effects	Boot SE	Bootstrap 95%CI		Relative mediation efect
			Boot LLCI	Boot ULCI	
Total efect	.84	.05	.74	.94	
Direct effect	.65	.06	.54	.76	
Indirect efect	.19	.03	.13	.26	22.62%

*Note. All variables in the model have been standardized*.

### A Moderated Mediation Model Test of Self-Control

Next, the PROCESS macro (*Model 7*) (Hayes, 2013) was conducted to test potential mediating models of regulation. After controlling for gender and age, the results (see *Table 5*) indicated that self-control signiicantly predicted psychological distress (β = -.13, *p <* .001) and that the perceived stress and self-control interaction term significantly predicted psychological distress (β = -.03, *p* < .001), suggesting that self-control moderates the prediction of psychological distress by perceived stress.

**Figure 2.**
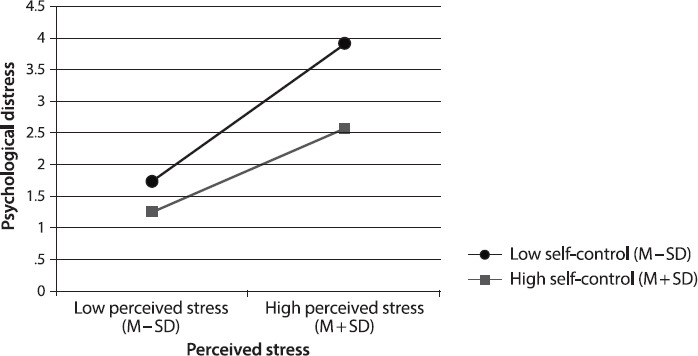
Moderating effect of self-control on perceived stress and psychological distress

Finally, to gain insight into the substance of the moderating effect of self-control, a simple slope test was conducted. For young adults with low levels of self-control, perceived stress was a significant positive predictor of psychological distress (β = .028, t = 16.865, *p <* .001). For young adults with high levels of self-control, perceived stress remained a signiicant positive predictor of psychological distress, with a weakened predictive effect (β = .026, t = 11.396, *p <* .001), suggesting that a high self-control personality would weaken the effect of having perceived stress of multiple psychological distress. The results are shown in *Figure 2*, where the positive correlation between perceived stress and psychological distress was stronger when the level of self-control was lower. Thus, young adults’ self-control moderates the link between perceived stress and psychological distress, which supports H3.

**Table 5:** Moderated Mediation Model Tests

	Model 1 (Psychological distress)		Model 2 (SABA)	
	β	t	β	t
Gender	.04	.51	.22	.97
Age	.09	1.64	-.32	-2.23^a^
Perceived stress	.38	16.29^c^	.65	11.46^c^
Self-control	-.13	-8.40^c^			
Perceived stress χ Self-control	-.03	-7.09^c^			
Psychological distress			.42	7.04^c^
*R* ^*2*^	.28		.15	
*F*	146.16^c^		86.61^c^	

*Notes. All variables in the model have been standardized. N = 1911. Gender is a dummy variable, encoding 0 = female and 1 =male. SABA = Smartphone Application-Based Addiction*. ^*a*^ *= p <.05;* ^*b*^ *= p <.01;*^*c*^ *= p <.001*.

## Discussion

The purpose of this study was to examine how perceived stress relates to SABA among young adults, particularly considering the mediating factor of psychological distress and the moderating factor of self-control. The results indicated that perceived stress was a significant and positive predictor of SABA. Furthermore, psychological distress partially mediated the relationship between perceived stress and SABA. Notably, for individuals exhibiting lower self-control levels, psychological distress was more susceptible to the influences of perceived stress.

### Theoretical Implications

The results of this study contribute to the existing literature on the psychological mechanisms underlying smartphone addiction.

Firstly, our results underscore that perceived stress signiicantly predicts SABA. This supports past findings that suggest stress may push individuals toward unhelpful coping strategies, such as excessive utilization of smartphones (Elhai et al., 2017; Elhai et al., 2019). Stress and Coping Theory (Lazarus & Folkman, 1984), which suggests that stressed individuals frequently resort to engagement with speciic smart-phone applications (*e.g*., social messaging, short-video, mobile gaming) as coping, which can inadvertently foster app-based compulsive behaviors.

Secondly, our findings confirm that psychological distress partially mediates the relationship between perceived stress and SABA, with distress acting as an emotional intermediary that amplifies stress into addictive tendencies. Theoretically, while both constructs involve negative experiences, perceived stress is a precursor appraisal that can trigger distress without necessarily causing it directly; distress, however, manifests as measurable emotional turmoil, making it a key mediator per the Self-Medication Hypothesis (Khantzian, 1997). Methodologically, the strong correlation does not imply redundancy, as regression analyses show independent predictive paths (e.g., stress directly predicts distress, β = .47, p < .001), supporting distinct constructs. This mediation underscores that interventions targeting distress could disrupt the pathway from stress to SABA more efectively than addressing stress alone.

While our study did not differentiate between specific application types, the Self-Medication Hypothesis provides a framework for speculating which applications are likely used as coping strategies for psychological distress. First, short-video platforms (e.g., Douyin/TikTok) offer a continuous, algorithmically-driven stream of novel and emotionally engaging content, which can act as a powerful distractor from stressful thoughts and negative afect, essentially serving as a digital paciier. Second, social media applications (e.g., WeChat, Weibo) may be used to self-medicate feelings of loneliness or low self-worth by seeking social validation through ‘likes” and comments, providing temporary relief from distress. Third, immersive mobile games can ofer a sense of control, achievement, and escape from the pressures of reality, making them an attractive coping tool for individuals experiencing stress-induced helplessness. Future research should therefore move beyond a general measure of SABA and investigate the diferential relationships between stress, psychological distress, and addiction to specific categories of applications. This would provide a more granular understanding of the problem and allow for more targeted interventions.

Thirdly, our research offers empirical validation for the Moderation-Mediation Model, underscoring self-control as a pivotal moderating variable. This investigation indicates that individuals exhibiting lower levels of self-control are more vulnerable to the negative repercussions of perceived stress on psychological distress. This conclusion supports the Self-Regulation Theory (Van Horn, 1995), which asserts that those with greater self-control are more skilled at coping with their stress and emotions, while steering clear of maladaptive actions. In contrast, those with lower self-control may be at an increased risk of experiencing intensiied psychological distress during stressful circumstances, thereby elevating their likelihood of SABA. The moderating role of self-control further emphasizes the significance of individual diferences in the susceptibility to smartphone addiction.

### Practical Implications

The findings derived from this research present numerous practical ramifications for interventions designed to diminish SABA among young adults in China. Interventions must be speciically adapted to the nuances of the Chinese digital landscape. Considering the pervasive nature of “super-apps”, methods aimed only at minimizing overall screen time may be unrealistic and counterproductive. A more sophisticated approach is necessary, instructing young adults to navigate these intricate digital ecosystems with mindfulness. Cognitive-behavioral techniques could be modified to assist individuals in recognizing and managing triggers associated with speciic app functionalities — such as compulsive browsing of integrated e-commerce platforms or excessive interaction with social media feeds — rather than entirely avoiding the necessary applications.

Primarily, it is imperative to prioritize stress management initiatives to aid individuals in cultivating robust coping mechanisms for handling perceived stress. Practices including mindfulness stress reduction and cognitive-behavioral approaches have been efective at reducing stress and its psychological symptoms (Grossman et al., 2004; Hofmann et al., 2012). Furthermore, stress management programs and psychological counseling may be effective in mitigating the impact of stress on appbased coping use (e.g., short-video scrolling, social messaging loops, mobile gaming) (Park & Lee, 2012).

Secondly, intervention strategies aimed at addressing SABA should integrate elements intended to enhance self-control. Empirical studies suggest that interventions prioritizing the augmentation of self-control can substantially reduce addictive tendencies (Muraven, 2010). Moreover, enhancing self-control via cognitive-behavioral techniques may aid individuals in navigating stress more proiciently and mitigating their reliance on smartphones (Hofmann et al., 2012).

Thirdly, in light of the partial mediating influence of psychological distress, it is crucial to tackle the foundational psychological concerns prevalent among young adults. Conducting screenings for indicators of anxiety, depression, and other types of psychological distress within educational settings and workplaces can facilitate the identiication of individuals susceptible to SABA. Ensuring access to counseling services and mental health support can alleviate the psychological distress that propels individuals towards SABA, particularly for those with low self-control.

### Future Research

Building on the current indings, future research should adopt longitudinal designs to better ascertain the causal pathways between perceived stress, psychological distress, self-control, and SABA. Such studies can illuminate how these relationships evolve over time and identify critical periods for intervention.

We need to delve deeper into cultural factors. Comparative studies across different cultural contexts can reveal how cultural norms and values inluence stress responses and coping mechanisms, ofering tailored intervention strategies that resonate with speciic cultural settings.

Exploring further moderating variables may also yield beneficial insights. Factors such as social support, personality traits, and previous experiences with stress and addiction could provide deeper insights into the individual diferences that shape the stress-SABA relationship. Identifying these moderating inluences can help reine intervention programs to target individuals at higher risk more efectively.

Critically, future investigations must address the application-speciic nature of SABA by examining which categories of applications (social media, gaming, short-video platforms, or entertainment streaming) serve as primary coping mechanisms for different types of psychological distress. Mixed-methods approaches combining quantitative usage data with qualitative interviews about application choice during distressing moments would provide invaluable insights into the content-centric nature of SABA.

## Conclusion

To sum up, this study contributes crucial knowledge about the intricate dynamics between perceived stress, psychological distress, self-control, and SABA among young adults. The results highlight the necessity of tackling psychological distress and improving self-control in programs designed to reduce smartphone addiction. Subsequent investigations ought to persist in examining these associations across varied demographic groups and utilize longitudinal methodologies to ascertain causal links.

## Limitations

Notwithstanding its merits, this research is not devoid of limitations. The cross-sectional framework restricts the capacity to deduce causal linkages. Although our results indicate noteworthy correlations, longitudinal investigations are essential to ascertain the directional impacts among perceived stress, psychological distress, self-control, and SABA. In addition, our reliance on metrics provided by individuals could foster biases, including social desirability and recall bias. Future research could incorporate objective measures of smartphone application use and physiological indicators of stress (van Deursen et al., 2015).

Further, the inquiry emphasized young adults in China, possibly restricting the extent to which indings are relevant in diverse cultural landscapes. Future cross-cultural studies should move beyond simple comparisons of prevalence rates to investigate how the structural diferences in digital ecosystems inluence the psychological mechanisms of addiction. We need cross-cultural studies to investigate the validity of the observed relationships in diverse cultural settings (Billieux et al., 2015).

## References

[ref1] AlSaif, H.I., Alhozaimi, Z.A., Alrashed, A.S., Alanazi, K.S., Alshibani, M.G., Almigbal, T.H., Alsaad, S.M., Alrasheed, A.A., & Alosaimi, F.D. (2023). Is there an association between increased stress and smartphone addiction? Insights from a study on medical students from Saudi Arabia during the COVID-19 pandemic. Medicina, 59(8), 1501. 10.3390/medicina59081501 37629791 PMC10456896

[ref2] Baumeister, R.F., Vohs, K.D., & Tice, D.M. (2007). The strength model of self-control. Current Directions in Psychological Science, 16(6), 351-355. 10.1111/j.1467-8721.2007.00534.x

[ref3] Baxter, A.J., Charlson, F.J., Cheng, H.G., Shidhaye, R., Ferrari, A.J., & Whiteford, H.A. (2016). Prevalence of mental, neurological, and substance use disorders in China and India: A systematic analysis. The Lancet Psychiatry, 3(9), 832-841. 10.1016/S2215-0366(16)30139-0 27528097

[ref4] Billieux, J., Maurage, P., Lopez-Fernandez, O., Kuss, D.J., & Griffiths, M.D. (2015). Can disordered mobile phone use be considered a behavioral addiction? An update on current evidence and a comprehensive model for future research. Current Addiction Reports, 2(2), 156-162. 10.1007/s40429-015-0054-y

[ref5] Chen, L., Yan, Z., Tang, W., Yang, F., Xie, X., & He, J. (2016). Mobile phone addiction levels and negative emotions among Chinese young adults: The mediating role of interpersonal problems. Computers in Human Behavior, 55, 856-866. 10.1016/j.chb.2015.10.030

[ref6] Chen, Y.L., & Kuo, P.H. (2020). Effects of perceived stress and resilience on suicidal behaviors in early adolescents. European Child & Adolescent Psychiatry, 29(6), 861-870. 10.1007/s00787-019-01401-w 31492979

[ref7] Cohen, S., Kamarck, T., & Mermelstein, R. (1983). A global measure of perceived stress. Journal of Health and Social Behavior, 24(4), 385-396. 10.2307/2136404 6668417

[ref8] Csibi, S., Griffiths, M.D., Cook, B., Demetrovics, Z., & Szabo, A. (2018). The psychometric properties of the Smartphone Application-Based Addiction Scale (SABAS). International Journal of Mental Health and Addiction, 16(2), 393-403. 10.1007/s11469-017-9787-2 29670500 PMC5897481

[ref9] De Ridder, D.T.D., Lensvelt-Mulders, G., Finkenauer, C., Stok, F.M., & Baumeister, R.F. (2012). Taking stock of self-control: A meta-analysis of how trait self-control relates to a wide range of behaviors. Personality and Social Psychology Review, 16(1), 76-99. 10.1177/1088868311418749 21878607

[ref10] Elayoubi, J., Haley, W.E., Roth, D.L., Cushman, M., Sheehan, O.C., Howard, V.J., Hladek, M.D., & Huelu-er, G. (2023). Associations of perceived stress, depressive symptoms, and caregiving with inflammation: A longitudinal study. International Psychogeriatrics, 35(2), 95-105. 10.1017/S1041610222000370 35543307 PMC11804796

[ref11] Elhai, J.D., Levine, J.C., Dvorak, R.D., & Hall, B.J. (2017). Non-social features of smartphone use are most related to depression, anxiety and problematic smartphone use. Computers in Human Behavior, 69, 75-82. 10.1016/jxhb.2016.12.023

[ref12] Elhai, J.D., Rozgonjuk, D., Yildirim, C., Alghraibeh, A.M., & Alafnan, A.A. (2019). Worry and anger are associated with latent classes of problematic smartphone use severity among college students. Journal of Affective Disorders, 246, 209-216. 10.1016/j.jad.2018.12.047 30583147

[ref13] Flynn, E.A., Thériault, é.R., & Williams, S.R. (2020). The use of smartphones to cope with stress in university students: Helpful or harmful? Journal of Technology in Behavioral Science, 5(2), 171-177. 10.1007/s41347-019-00125-7

[ref14] Geng, Y.G., Gu, J.J., Wang, J., & Zhang, R.P. (2021). Smartphone addiction and depression, anxiety: The role of bedtime procrastination and self-control. Journal of Affective Disorders, 293, 415-421. 10.1016/j.jad.2021.06.062 34246950

[ref15] Glowacz, F., & Schmits, E. (2020). Psychological distress during the COVID-19 lockdown: The young adults most at risk. Psychiatry Research, 293, 113486. 10.1016/j.psychres.2020.113486 33007682 PMC7518205

[ref16] Grossman, P., Niemann, L., Schmidt, S., & Walach, H. (2004). Mindfulness-based stress reduction and health benefits. Journal of Psychosomatic Research, 57(1), 35-43. 10.1016/S0022-3999(03)00573-7 15256293

[ref17] Guan, Q., Dong, H., Zhang, Z., Guo, Z., Lin, Z., Niu, H., Wu, Y., & Hou, H. (2024). The mediating effect of perceived stress on the relationship between big ive personality traits and suboptimal health status in Chinese population: A nationwide survey in the framework of predictive, preventive, and personalized medicine. EPMA Journal, 15(1), 25-38. 10.1007/s13167-023-00349-x 38463623 PMC10923761

[ref18] Hayes, A.F. (2013). Introduction to mediation, moderation, and conditional process analysis: A regression-based approach. The Guilford Press.

[ref19] Hofmann, S.G., Asnaani, A., Vonk, I.J.J., Sawyer, A.T., & Fang, A. (2012). The efficacy of cognitive behavioral therapy: A review of meta-analyses. Cognitive Therapy and Research, 36(5), 427-440. 10.1007/s10608-012-9476-1 23459093 PMC3584580

[ref20] Hofmann, W., Schmeichel, B.J., & Baddeley, A.D. (2012). Executive functions and self-regulation. Trends in Cognitive Sciences, 16(3), 174-180. 10.1016/j.tics.2012.01.006 22336729

[ref21] Hong, Y.P., Yeom, Y.O., & Lim, M.H. (2021). Relationships between smartphone addiction and smart-phone usage types, depression, ADHD, stress, interpersonal problems, and parenting attitude with middle school students. Journal of Korean Medical Science, 36(19), Article e129. 10.3346/jkms.2021.36.e129 PMC8129617 34002549

[ref22] Irimiás, A., Csordás, T., Kiss, K., & Michalkó, G. (2021). Aggregated roles of smartphones in young adults’ leisure and well-being: A diary study. Sustainability, 13(8), Article 4133. 10.3390/su13084133

[ref23] Kardefelt-Winther, D. (2014). A conceptual and methodological critique of internet addiction research: Towards a model of compensatory internet use. Computers in Human Behavior, 31, 351-354. 10.1016/j.chb.2013.10.059

[ref24] KayiÇ, A.R. (2022). Mindfulness, impulsivity and psychological distress: Th e mediation role of smartphone addiction. British Journal of Guidance & Counselling, 50(5), 791-804. 10.1080/03069885.2022.2046255

[ref25] Khantzian, E.J. (1997). The self-medication hypothesis of substance use disorders: A reconsideration and recent applications. Harvard Review of Psychiatry, 4(5), 231-244. 10.3109/10673229709030550 9385000

[ref26] Kroenke, K., Spitzer, R.L., Williams, J.B.W., & Löwe, B. (2009). An ultra-brief screening scale for anxiety and depression: The PHQ-4. Psychosomatics, 50(6), 613-621. 10.1016/S0033-3182(09)70864-3 19996233

[ref27] Lane, J.A., Leibert, T.W., & Goka-Dubose, E. (2017). The impact of life transition on emerging adult attachment, social support, and well-being: A multiple-group comparison. Journal of Counseling & Development, 95(4), 378-388. 10.1002/jcad.12153

[ref28] Law, M.-Y., & Yap, J.-J. (2022). Psychological distress and smartphone addiction: Does perceived social support make a difference? International Journal of Emerging Trends in Social Sciences, 12(2), 22-32. 10.55217/103.v12i2.550

[ref29] Lazarus, R., & Folkman, S. (1984) Stress, appraisal, and coping. Springer.

[ref30] Li, Y., Li, G., Liu, L., & Wu, H. (2020). Correlations between mobile phone addiction and anxiety, depression, impulsivity, and poor sleep quality among college students: A systematic review and meta-analysis. Journal of Behavioral Addictions, 9(3), 551-571. 10.1556/2006.2020.00057 32903205 PMC8943681

[ref31] Lian, S.L., Sun, X.J., Niu, G.F., Yang, X.J., Zhou, Z.K., & Yang, C. (2021). Mobile phone addiction and psychological distress among Chinese adolescents: The mediating role of rumination and moderating role of the capacity to be alone. Journal of Affective Disorders, 279, 701-710. 10.1016/j.jad.2020.10.005 33197839 PMC7539895

[ref32] Liu, H., Novotny, J.S., & Vachova, L. (2022). The effect of mobile phone addiction on perceived stress and mediating role of ruminations: Evidence from Chinese and Czech university students. Frontiers in Psychology, 13, Article 1057544. 10.3389/fpsyg.2022.1057544 PMC9806227 36600696

[ref33] Luo, Y., Zhang, Y., Sun, X., Dong, J., Wu, J., & Lin, X. (2022). Mediating effect of self-control in the relationship between psychological distress and food addiction among college students. Appetite, 179, 106278. 10.1016/j.appet.2022.106278 35988799

[ref34] Morean, M.E., DeMartini, K.S., Leeman, R.F., Pearlson, G.D., Anticevic, A., Krishnan-Sarin, S., Krys-tal, J.H., & O’Malley, S.S. (2014). Psychometrically improved, abbreviated versions of three classic measures of impulsivity and self-control. Psychological Assessment, 26(3), 1003-1020. 10.1037/pas0000003 24885848 PMC4152397

[ref35] Muraven, M. (2010). Building self-control strength: Practicing self-control leads to improved self-control performance. Journal of Experimental Social Psychology, 46(2), 465-468. 10.1016/j.jesp.2009.12.011 20401323 PMC2855143

[ref36] Olson, J.A., Sandra, D.A., Colucci, é.S., Al Bikaii, A., Chmoulevitch, D., Nahas, J., Raz, A., & Veissière, S. P. L. (2022). Smartphone addiction is increasing across the world: A meta-analysis of 24 countries. Computers in Human Behavior, 129, 107138. 10.1016/jxhb.2021.107138

[ref37] Park, N., & Lee, H. (2012). Social implications of smartphone use: Korean college students’ smartphone use and psychological well-being. Cyberpsychology, Behavior, and Social Networking, 15(9), 491497. 10.1089/cyber.2011.0580 22817650

[ref38] Podsakoff, P.M., MacKenzie, S.B., Lee, J.Y., & Podsakoff, N.P. (2003). Common method biases in behavioral research: A critical review of the literature and recommended remedies. Journal of Applied Psychology, 88(5), 879-903. 10.1037/0021-9010.88.5.879 14516251

[ref39] Ridner, S.H. (2004). Psychological distress: Concept analysis. Journal of Advanced Nursing, 45(5), 536-545. 10.1046/j.1365-2648.2003.02938.x 15009358

[ref40] Shen, B., Fan, W., Shaojing, S., & and Liu, Y. (2021). Chinese adolescents’ academic stress and smartphone addiction: A moderated-mediation model. Journal of Broadcasting & Electronic Media, 65(5), 724-740. 10.1080/08838151.2021.2014842

[ref41] Shruthi, M. N., Veena, V., & Seeri, J. S. (2023). Prevalence of psychological distress and perceived stress among nursing staff in a tertiary care center, Bengaluru. MRIMS Journal of Health Sciences, 11(1), 41-47. 10.4103/mjhs.mjhs_28_22

[ref42] Sohn, S.Y., Rees, P., Wildridge, B., Kalk, N.J., & Carter, B. (2019). Prevalence of problematic smart-phone usage and associated mental health outcomes amongst children and young people: A systematic review, meta-analysis and GRADE of the evidence. BMC Psychiatry, 19(1), 356. 10.1186/s12888-019-2350-x 31779637 PMC6883663

[ref43] Song, T.-J., & Zhao, H. (2025). Psychometric properties and measurement invariance across gender of the Chinese version of the Smartphone Application-Based Addiction Scale (SABAS) among Chinese college students. PLoS One, 20(5). 10.1371/journal.pone.0323215 PMC12094724 40397890

[ref44] Tao, L., Li-mei, C., Li-xia, Q., & Shui-yuan, X. (2021). Reliability and validity of chinese version of Brief Self-Control Scale. Chinese Journal of Clinical Psychology, (1), 83-86. 10.16128/j.cnki.1005-3611.2021.01.017

[ref45] Tng, G.Y.Q., & and Yang, H. (2024). Nuanced relationships between indices of smartphone use and psychological distress: distinguishing problematic smartphone use, phone checking, and screen time. Behaviour & Information Technology, 43(5), 956-969. 10.1080/0144929X.2023.2196573

[ref46] van Deursen, A.J.A.M., Bolle, C.L., Hegner, S.M., & Kommers, P.A.M. (2015). Modeling habitual and addictive smartphone behavior. Computers in Human Behavior, 45, 411-420. 10.1016/j.chb.2014.12.039

[ref47] Van Horn, D.H.A. (1995). Losing control: How and why people fail at self-regulation. Clinical Psychology Review, 15(4), 367-368. 10.1016/0272-7358(95)90149-3

[ref48] Varma, P., Junge, M., Meaklim, H., & Jackson, M.L. (2021). Younger people are more vulnerable to stress, anxiety and depression during COVID-19 pandemic: A global cross-sectional survey. Progress in Neuro-Psychopharmacology & Biological Psychiatry, 109, 110236. 10.1016/j.pnpbp.2020.110236 33373680 PMC7834119

[ref49] Veeramachaneni, K., Slavish, D.C., Dietch, J.R., Kelly, K., & Taylor, D.J. (2019). Intraindividual variability in sleep and perceived stress in young adults. Sleep Health, 5(6), 572-579. 10.1016/j.sleh.2019.07.009 31575485 PMC6917884

[ref50] Vujic, A., & Szabo, A. (2022). Hedonic use, stress, and life satisfaction as predictors of smartphone addiction. Addictive Behaviors Reports, 15, 100411. 10.1016/j.abrep.2022.100411 35746955 PMC9210358

[ref51] Wang, Y., & Wang, P. (2019). Perceived stress and psychological distress among Chinese physicians: The mediating role of coping style. Medicine, 98(23), e15950. 10.1097/MD.0000000000015950 31169719 PMC6571215

[ref52] Whiteford, H.A., Degenhardt, L., Rehm, J., Baxter, A.J., Ferrari, A.J., Erskine, H.E., Charlson, F.J., Norman, R.E., Flaxman, A.D., Johns, N., Burstein, R., Murray, C.J., & Vos, T. (2013). Global burden of disease attributable to mental and substance use disorders: findings from the Global Burden of Disease Study 2010. The Lancet, 382(9904), 1575-1586. 10.1016/s0140-6736(13)61611-6 23993280

[ref53] Xu, Y., Zhang, R., Zhou, Z., Fan, J., Liang, J., Cai, L., Peng, L., Ren, F., & Lin, W. (2021). Parental psychological distress and attitudes towards COVID-19 vaccination: A cross-sectional survey in Shenzhen, China. Journal of Affective Disorders, 292, 552-558. 10.1016/j.jad.2021.06.003 34147967 PMC8179837

[ref54] Yu, B., Yue, G., & Liu, H. (2013). The strength model of self-control. Advances in Psychological Science, 21(7), 1272-1282. 10.3724/SPJ.1042.2013.01272

[ref55] Zhang, S., Tao, S., Zhang, Y.-L., Zhou, J., Wei, J., Chen, M., Hu, Q., Zheng, H., & Wang, Z.-L. (2025). Examining the spectrum of problematic online behaviors in Chinese adolescents: A network analysis of smartphone, gaming, and social media use. Computers in Human Behavior, 167, 108611. 10.1016/j.chb.2025.108611

[ref56] Zhang, W., Pu, J., He, R., Yu, M., Xu, L., He, X., Chen, Z., Gan, Z., Liu, K., Tan, Y., & Xiang, B. (2022). Demographic characteristics, family environment and psychosocial factors affecting internet addiction in Chinese adolescents. Journal of Affective Disorders, 315, 130-138. 10.1016/j. 35901990

